# Oxidative Stress in Patients Undergoing Peritoneal Dialysis: A Current Review of the Literature

**DOI:** 10.1155/2017/3494867

**Published:** 2017-12-27

**Authors:** Vassilios Liakopoulos, Stefanos Roumeliotis, Xenia Gorny, Theodoros Eleftheriadis, Peter R. Mertens

**Affiliations:** ^1^Division of Nephrology and Hypertension, 1st Department of Internal Medicine, AHEPA Hospital, School of Medicine, Aristotle University of Thessaloniki, Thessaloniki, Greece; ^2^Clinic of Nephrology and Hypertension, Diabetes and Endocrinology, Otto-von-Guericke University Magdeburg, Magdeburg, Germany

## Abstract

Peritoneal dialysis (PD) patients manifest excessive oxidative stress (OS) compared to the general population and predialysis chronic kidney disease patients, mainly due to the composition of the PD solution (high-glucose content, low pH, elevated osmolality, increased lactate concentration and glucose degradation products). However, PD could be considered a more biocompatible form of dialysis compared to hemodialysis (HD), since several studies showed that the latter results in an excess accumulation of oxidative products and loss of antioxidants. OS in PD is tightly linked with chronic inflammation, atherogenesis, peritoneal fibrosis, and loss of residual renal function. Although exogenous supplementation of antioxidants, such as vitamins E and C, N-acetylcysteine, and carotenoids, in some cases showed potential beneficial effects in PD patients, relevant recommendations have not been yet adopted in everyday clinical practice.

## 1. Introduction

Oxidative stress (OS) is defined as the tissue injury and the systemic damage caused by disrupted balance between oxidative molecules and insufficient antioxidant defense mechanisms [1, 2]. Among the plethora of oxidative products, reactive oxygen species (ROS) and nitric oxide (NO) are the most common, while antioxidants can be molecules either endogenously synthesized or exogenously administered. The main targets of OS-induced damage are proteins, carbohydrates, lipids, and nucleic acids (DNA). Chronic kidney disease (CKD) is characterized by enhanced oxidation status of proteins, lipids and DNA, and subsequent tissue and organ injury. OS is evident even in the early stages of CKD, progresses along with the deterioration of renal function, and is further exacerbated in patients undergoing dialysis. There is a growing body of evidence showing that OS is a crucial promoter of atherosclerosis in end-stage renal disease (ESRD) [2–4]. Moreover, OS is of paramount relevance for the chronic inflammation state and ensuing fibrosis of the peritoneum in patients undergoing peritoneal dialysis (PD). It is also related to residual renal function (RRF). On the other hand, antioxidant supplementation is an emerging strategy to counteract OS with the potential to preserve peritoneal function. However, although OS in hemodialysis (HD) has been thoroughly studied during the past decade, the data regarding the pathogenesis, role, and predictive value of OS in PD patients is still limited but constantly growing.

## 2. OS Status in PD

### 2.1. Pathophysiological Mechanism

Both HD and PD are accompanied by enhanced OS, although underlying mechanisms are considered to differ. In HD, the formation of oxidative products is associated with the type of dialysis membranes, use of heparin, intravenous iron administration, and activation of platelets and leukocytes. In contrast, the composition of the PD solution (low pH, elevated osmolality, increased lactate concentration, and glucose degradation products) is responsible for accumulation of oxidative products [5]. In vitro studies showed that when cultured human mesothelial cells are exposed to dextrose or icodextrin dialysates for one hour, it produces ROS, increasing thus OS [6]. In line with these results, the limited “biocompatibility” of the peritoneal dialysis solution results in accumulation of ROS [7]. Huh et al. suggested that accumulation of glucose degradation molecules in glucose-containing peritoneal solutions may trigger the onset and development of OS [8]. Moreover, the chronic inflammation status that accompanies the long-used peritoneum causes dramatic structural and functional changes in the peritoneal membrane and contributes to enhanced OS [9, 10]. Due to the bioincompatibility of the conventional PD solution, increased production of serum inflammation markers such as interleukin-6 (IL-6) is triggered early, even within the first hour after the initiation of PD treatment [11, 12]. Data from peritoneal biopsies in healthy controls and CKD patients, before commencement of PD and after short- and long-term (>18 months) PD treatment, showed that peritoneal nitric oxide synthase (NOS) activity (the enzyme that triggers the production of the prooxidant NO) was increased five times more in the long-term PD group compared to controls. Moreover, this overexpression of NOS in long-term PD patients was associated with enhanced calcification of the peritoneal membrane, increased activity of vascular endothelial growth factor (VEGF), and accumulation of advanced glycation end-products (AGEs). Therefore, it is suggested that overproduction of NO, a well-known prooxidative marker that causes endothelial damage, may underlie the structural alterations of the peritoneal membrane seen in chronic PD patients [13]. Similarly, Miyata et al. showed that breakdown of conventional PD solutions leads to formation of oxidation carbonyl products and subsequently results in dramatic changes in the structure and function of peritoneal proteins and accumulation of AGEs [14]. Thus, it can be concluded that in PD patients, the enhanced OS status is linked with the peritoneal solution composition.

### 2.2. Comparison of OS Status in Healthy Population versus PD Patients

PD patients manifest excessive OS compared to the general population and predialysis CKD patients. Chronic inflammation and OS markers (as assessed by glutathione peroxidase and superoxide activity, total antioxidant capacity-TAC, malondialdehyde-MDA levels, and protein carbonyl formation) in blood, urine, and peritoneal fluid were significantly elevated in PD patients, compared to healthy controls [15]. Another study showed that serum levels of several well-established OS markers such as thiobarbituric acid-reactive substances (TBARS), MDA, AOPPs, AGEs, and asymmetric dimethylarginine (ADMA) were significantly increased in PD patients compared to healthy age- and gender-matched controls [16]. Schmidt et al. [17] sought to determine the degree of OS severity in PD compared to the general population and demonstrated that ADMA levels were five times higher in PD patients than in healthy controls. Terawaki et al. showed that oxidized albumin—another marker of OS status—was significantly increased in 21 chronic continuous ambulatory peritoneal dialysis (CAPD) patients compared to healthy controls and was tightly associated with blood urea nitrogen but not with urine volume or serum concentration of *β*2-microglobulin. These authors concluded that serum albumin oxidation might be promoted by small and middle-sized uremic toxins in chronic stable CAPD patients [18]. Another study showed that CAPD is a state of enhanced OS in lipids (indicated by high-plasma TBARS levels), red blood cells (RBCs), and plasma (assessed by decreased glutathione to oxidized glutathione ratio—GSH/GSSG) when compared to matched healthy controls [19]. Excessive lipid peroxidation in CAPD patients compared to healthy individuals was also reported in another study by Zima et al. in 1996. The authors found higher levels of MDA in both serum and RBCs in CAPD patients versus healthy controls [20]. A recent study confirmed that serum levels of NO and MDA were significantly higher in PD patients compared to healthy controls [21]. Similar results regarding the enhanced lipid peroxidation status in PD patients were published by Nourooz-Zadeh et al. Plasma lipid hydroperoxides (ROOH) and TBARS were determined in 12 CKD predialysis, 12 CAPD, 36 chronic HD patients, and 20 healthy controls. Although serum TBARS concentrations were similar in control and all three renal impairment groups, serum ROOHs were significantly increased in the HD group compared to controls. CAPD patients had slightly higher but not significantly increased plasma ROOH levels than controls [22]. Uzum et al. determined serum concentrations of oxidant and antioxidant molecules in PD, HD, and healthy controls and showed that the control group presented significantly lower levels of the oxidant MDA and significantly higher levels of the antioxidant superoxide dismutase (SOD) and vitamin E than both HD and PD patients [23]. MDA serum levels were found significantly higher in PD patients compared to healthy controls by Sundl et al. [24]. Another study showed that the serum levels of 8-hydroxy 2-deoxyguanosine (8-OHdG), a well-established marker of oxidative damage in DNA, was significantly higher in PD patients when compared to predialysis CKD patients and healthy age- and sex-matched controls. Moreover, PD patients presented a pronounced deficiency of antioxidants, as expressed by lower serum levels of ascorbic acid, vitamin E, and glutathione when compared to the control group [25]. Zwolinska et al. found that children on PD had reduced activity of GSH in RBCs and plasma and significantly decreased levels of antioxidant vitamins A, C, and E—due to loss of these vitamins into the ultrafiltrate—compared to age-matched healthy controls [26].

The above-mentioned studies indicate that PD patients are characterized by increased formation of oxidative molecules and loss of antioxidants compared to the general population and predialysis CKD patients.

### 2.3. High-Glucose Concentrations and AGEs Contained in the PD Solutions Are the Culprit of OS in PD

Although it is well-established that PD is a more biocompatible dialysis method than HD since the uremic toxins are excreted via the patient's peritoneal membrane—a natural filter—and not through a HD dialyzer, there are still issues of bioincompatibility due to the composition of the PD fluids. Commercially used PD fluids are characterized by increased glucose levels and osmolarity, low pH, and presence of lactate. During heat sterilization of the PD fluids, glucose degradation products (GDPs) are accumulated in the peritoneum and trigger the formation of AGEs, ROS, and advanced oxidation protein products (AOPPs) in excessive amounts leading to an increased OS status. A high OS status, increased systemic and local inflammation, apoptosis of all peritoneal cells, membrane fibrosis, and loss of ultrafiltration are characteristics of the chronic exposure of the peritoneal membrane to AGEs.

During PD, all peritoneal cells (mesothelial, endothelial, and white blood cells) are exposed to the hostile, high-glucose environment caused by glucose or its degradation products and undergo crucial structural and functional alterations. The OS caused by the glycemic environment leads to irreversible, severe DNA and protein damage, and subsequent apoptotic cell death [27, 28].

*In vitro* studies have demonstrated that mesothelial cell incubation with high-glucose PD fluids was accompanied by a rapid (within 1 minute) shrinkage of the cells and subsequent apoptotic necrosis after 2 hours [29]. Both *in vitro* and *in vivo* studies have repeatedly shown that the glucose directly causes time- and dose-dependent mitochondrial genomic damage of the mesothelial cells and subsequent cellular death. Both lipid and protein oxidation triggered by the formation of GDPs have been suggested as OS mediators leading to DNA damage [30–32]. In agreement with these results, peritoneal mesothelial cells were incubated at various glucose concentrations (5 mM, 84 mM, 138 mM, and 236 mM) with and without coadministration of N-acetylcysteine (NAC), a well-established antioxidant ROS scavenger. Exposure of the mesothelial cells to the high-glucose environment (236 mM) resulted in excessive formation and accumulation of ROS and lipid peroxides and subsequently severe mitochondrial DNA injury and disruption of the cell membrane. Administration of NAC inhibited the caspase-mediated OS and protected mitochondria and mesothelial cells from injury and apoptosis [33]. Similarly, other *in vitro* experiments have suggested that incubation of endothelial peritoneal cells with glucose-enriched PD fluids may lead to OS-derived cell death [34]. In agreement, *in vivo* studies have demonstrated that the AGEs accumulated by glucose degradation seem to trigger the oxidative response inducing cell necrosis of the peritoneal microvasculature. Although the exact mechanism underlying this process has not yet been fully elucidated, it is suggested that accumulation of ROS may be the culprit of endothelial apoptosis [35]. Moreover, AGEs were massively accumulated in the plasma of PD patients with longer dwell times. Likewise, leucocytes exposed to high glucose (4.25%) and GDP PD fluids may exhibit a caspase-dependent necrosis, strongly and directly associated with the concentration of GDPs [36].

Furthermore, a number of animal and human studies support that the continuous exposure of the peritoneal membrane to the high-glucose environment caused by peritoneal fluids leads not only to OS-mediated alterations of all peritoneal cells but also to activation of fibrogenic factors like transforming growth factor (TGF) beta1 [37, 38]. Finally, it has been reported that low-GDP PD fluids seem to preserve the integrity of peritoneal cells and protect from systemic inflammation and peritoneal cell apoptosis [38, 39]. Therefore, there is accumulating evidence supporting that formation of AGEs due to high-glucose PD fluids may be the culprit for the accelerated OS and the peritoneal membrane fibrosis reported in chronic PD patients [38, 39].

### 2.4. Comparison of OS Status in PD versus HD

Whether PD or HD patients exhibit higher oxidative cell damage remains unclear (Table 1). Some studies report a similar OS status for both modalities, while a few others demonstrate higher OS in PD than in HD patients. Six studies reported similar OS status in both modalities. Two studies showed that oxidized low-density cholesterol (ox-LDL) and OS-derived DNA damage did not differ significantly between dialysis patients treated with PD and HD [40, 41]. Samouilidou et al. showed that plasma levels of oxidized LDL cholesterol (ox-LDL) were significantly increased in PD patients compared to healthy individuals, but they did not differ significantly among PD and HD patients [40]. Similarly, Castoldi et al. reported no difference in serum levels of the oxidative biomarkers 8-OHdG and ox-LDL [41] and Filiopoulos et al. found similar OS status—as assessed by serum levels of TAC and SOD—in HD and PD patients [42]. Likewise, three other studies reported similar serum levels of the antioxidants TAC, SOD, CAT, and glutathione in HD and CAPD patients [43–45].

Only three studies reported higher OS status in PD versus HD patients. Al-Hweish et al. found that plasma levels of myeloperoxidase (MPO) were significantly elevated in CAPD patients than in patients treated with HD [46], and Taylor et al. reported increased lipid peroxidation status in patients undergoing CAPD compared to patients undergoing HD [47]. In agreement with these results, McGrath et al. found that, compared to HD, patients treated with CAPD presented higher plasma levels of lipid peroxidation—assessed by MDA—and increased RBC membrane fluidity [48]. The underlying mechanism explaining this finding could possibly be the high-serum levels of AGEs, triggered by the high-glucose environment caused by PD fluids [49].

On the contrary, there is a growing body of evidence supporting that HD results in higher degree of oxidative product accumulation and loss of antioxidants, compared to PD. Kielstein et al. showed that plasma ADMA levels in HD patients were sixfold higher than those in healthy controls but, in contrast, PD patients presented significantly lower ADMA concentrations compared with the HD group [50]. Two separate studies [51, 52] showed decreased levels of plasma F2-isoprostanes, a novel clinical OS biomarker, in CAPD patients compared with patients on HD. Another study showed that RBC membrane susceptibility to lipid peroxidation was significantly increased in patients undergoing HD treatment compared to CAPD patients. These investigators hypothesized that in contrast to HD, the well-established favorable impact of CAPD on the anemia status of ESRD patients might be a result of lower OS [53]. Similarly, Usberti et al. examined the oxidative damage, assessed by plasma levels of ROS, MDA, vitamin E, and TAC in 16 PD and 55 HD patients. The authors found increased OS in HD compared to PD patients that was further exacerbated by HD sessions [54]. The rate of superoxide release from activated neutrophils is a novel but significant biomarker of uremia-induced OS and was found significantly increased in HD patients than in patients undergoing CAPD [55]. Accordingly, another study showed that circulating ADMA levels were significantly increased in HD when compared to PD patients [56]. Pawlak et al. measured plasma levels of Cu/Zn SOD—as an OS marker—as well as VEGF, a proatherogenic cytokine, in 20 healthy controls and 42 predialysis CKD, 25 HD, and 45 PD patients. Cu/Zn SOD and VEGF plasma levels were significantly lower in the control group than in the CKD group. HD patients presented higher levels of these markers compared to CKD and PD patients, indicating that PD might be more “biocompatible” than HD [57]. Likewise, Kayabasi et al. evaluated the plasma levels of the OS products MDA, GPx, and the antioxidant SOD in 32 maintenance HD, 39 CAPD patients, and 30 healthy controls and found that compared to PD patients, HD patients presented significantly lower levels of SOD and were more susceptible to OS development [58]. Yonova et al. showed that serum MDA and ox-LDL levels were significantly elevated in HD compared to CAPD patients [59]. In agreement with the previous studies, four other studies found that several OS markers were significantly higher in HD in comparison to PD patients [60–63]. Marques de Mattos et al. conducted a cross-sectional observational study to investigate the association between dialysis modality and OS status. Plasma levels of AOPPs, a biomarker of protein oxidation, were determined in 48 patients on HD, 20 on CAPD, and 17 healthy controls. HD patients presented significantly higher AOPP serum levels compared to controls. On the contrary, the CAPD and the control group presented similar levels of plasma AOPP. Serum AOPP levels were positively correlated with the plasma levels of triglycerides and inversely correlated with serum high-density lipoprotein (HDL) only in the HD group. No association was found between AOPP serum levels and markers of dyslipidemia in the PD group [62]. In a similar study by the same group, serum AOPPs and AGEs were also found significantly elevated in HD compared to PD patients [63]. Therefore, the authors concluded that HD may promote the formation of oxidative products in a greater degree than CAPD.

Several other studies compared the protein oxidation status in HD and PD patients: serum carbonyl proteins and TBARS were found excessively higher in HD patients than PD subjects in four occasions [52, 64–66]. This observation was attributed mainly to the better biocompatibility of PD as a dialysis method. A recent study in a large cohort of 303 dialysis patients (220 on HD 83 on PD) reported that HD modality and the length of time on dialysis were strong and independent predictors of serum levels of 8-OHdG, a marker of oxidative DNA damage. After a follow-up period of 31 months, plasma levels of 8-OHdG were associated with all-cause mortality only in the HD, not in the PD group [67]. Similarly, Ross et al. showed that both HD and PD patients were characterized by enhanced OS—assessed by increase serum levels of oxidized glutathione—compared to healthy controls. However, levels of oxidized glutathione were significantly higher in HD [68]. In order to investigate the redox state in CAPD and HD, Takayama et al. quantified the formation of glutathionyl hemoglobin in HD (before and after a HD session) and CAPD patients and found increased glutathionyl hemoglobin levels in HD compared to CAPD patients. In this study, the HD session did not influence the levels of glutathionyl hemoglobin [69]. Stępniewska et al. investigated the effect of dialysis modality on platelet antioxidant activity and found that catalase activity was significantly higher in PD compared to HD patients (0.82 in PD versus 0.52 before and 0.35 after HD treatment, resp.), while glutathione transferase activity was found 15 times higher in the PD than the HD group. Therefore, PD exhibited a beneficial effect on platelet OS compared to HD, acquiring probably a protective role against thrombotic episodes [70].

Several studies showed that concentrations of various antioxidants (such as vitamins A+C+E, TAC, Zn, Cu, and Se) in serum and erythrocytes and even platelets and antioxidant activity (SOD, CAT, and GPx) are significantly suppressed in HD patients compared to PD patients [52, 58–60, 70–73], while only two studies reported similar serum antioxidant status in both dialysis modalities [63, 64].

It can therefore be suggested that, compared to PD, HD patients manifest excessive oxidation of proteins, lipids, carbohydrates, and nucleic acids, while PD seems to better preserve the antioxidant defense mechanisms.

## 3. Inflammation, OS, and PD

Several investigators have repeatedly highlighted the strong association between dialysis and chronic inflammation [74, 75]. Borazan et al. showed that both HD and PD patients had significantly higher levels of inflammation biomarkers—like serum C—reactive protein (CRP), tumor necrosis factor-alpha (TNF-alpha), and IL-6—compared to age- and gender-matched healthy controls [76]. Serum levels of these markers did not differ significantly among the HD and the PD group. Another study also reported significantly higher serum concentrations of CRP, IL-6, and TNF-a in both HD and CAPD patients compared to healthy individuals but detected no difference between the two dialysis groups. Lipid abnormalities were more pronounced in the CAPD group compared to both control and HD group. In PD patients, plasma CRP levels were inversely correlated with serum concentration of HDL [62]. In line with these results, Filiopoulos et al. reported significantly increased serum levels of high-sensitive CRP in HD and PD patients compared to healthy controls. However, plasma concentrations of IL-6 and TNF-a did not differ significantly among dialysis patients and controls. The PD group had higher serum TNF-a levels than the HD patients, although without statistical significance. Moreover, in the PD group, TNF-a was strongly associated with dyslipidemia markers [42]. Another study showed that serum levels of circulating inflammatory markers such as IL-6, IL-8, and TNF-a were significantly higher in predialysis CKD patients than in healthy controls and even higher in CAPD patients. In the same study, the authors measured the same inflammatory biomarkers in the peritoneal effluent in two groups, stable CAPD patients and CAPD patients with peritonitis, and found significantly higher levels of all these markers in both serum and PD effluent in the peritonitis group. Furthermore, as peritonitis was clinically abated, their serum and dialysate levels were significantly decreased [77]. A recent prospective cohort study in 80 PD and 228 HD patients showed that the circulating IL-6 and CRP levels were significantly higher in the HD patients compared to the PD patients. Therefore the authors concluded that HD modality is associated with an increased inflammation status compared to PD [78]. In agreement with these results, Puchades et al. reported significantly lower levels of high-sensitivity CRP in PD patients compared to HD and predialysis CKD subjects [52]. This result was attributed to the higher prevalence of hypertension and DM2 in the HD and predialysis groups. In this study, there was no difference in serum levels of high-sensitivity CRP among PD patients and healthy control subjects. Serum CRP was also found higher in HD than PD patients by Guoa et al. and the inflammatory status in both dialysis groups was strongly and positively associated with plasma TBARS and negatively with the antioxidant elements zinc and selenium [60].

These results implicate that PD may represent a milder inflammatory condition compared to HD.

## 4. PD-Derived OS and Atheromatosis

There is a growing body of evidence indicating the tight linkage between enhanced OS, inflammation, atherosclerosis, and cardiovascular (CV) events in ESRD patients undergoing HD [79, 80]. OS in PD patients starts early, even during the first year of dialysis and causes endothelial damage, the first crucial step of atherosclerosis and CV disease. In PD patients, accumulation of AGEs, formation of oxidative products, loss of important antioxidants, and chronic inflammation are interrelated factors that lead to arterial stiffness, CV disease, and increased mortality [81, 82]. Although OS in PD patients is considered as a potential risk factor for serious clinical adverse outcomes, confirmatory data are very scarce. Jiang et al. found that excessive AGE production was strongly linked to CV morbidity in PD patients, independently of traditional risk factors [79]. Samouilidou et al. reported similar serum levels of ox-LDL for HD and PD patients and therefore concluded that both dialysis modalities are characterized by the same CV burden [40]. On the contrary, another recent cross-sectional study in 303 ESRD patients treated with either HD (*n* = 220) or PD (*n* = 83) showed that after a follow-up period of 31 months, serum 8-OHdG, a compound-reflecting oxidative DNA damage, was a strong predictor of all-cause mortality (HR 1.40) independently of various well-established risk factors such as age, body mass index, sex, comorbidity score, and dialysis vintage. When the patients were divided into subgroups according to dialysis modality, plasma 8-OHdG was tightly linked to all-cause mortality only in the HD group, but not in PD individuals [67]. In a cohort of 32 MHD and 39 CAPD patients, MDA was negatively correlated with cardiac function—assessed by ejection fraction—and the antioxidant SOD was significantly negatively associated with systolic and diastolic blood pressure, suggesting thus that dialysis-induced OS may play a role in the development of left ventricular hypertrophy [58]. Another study showed that OS in PD correlated strongly and independently with endothelial dysfunction and high-carotid intima-media thickness (cIMT)—a well-established surrogate marker of early subclinical atherosclerosis [16]. Klotho is an antiaging gene expressed mainly in the kidney. Plasma klotho levels are inversely associated with glomerular filtration rate and decrease along with progression of CKD. During the past decade, there is growing body of evidence showing that klotho deficiency directly leads to vascular calcification in renal failure [83]. A recent study reported that plasma levels of klotho were tightly and inversely linked with 8-isoprostane levels in a cohort of 78 PD patients, and therefore klotho deficiency may be associated with accelerated OS and vascular calcification development in these patients [84].

Although there is a growing body of evidence supporting that OS causes endothelial damage—the first key step to CVD—the data regarding the association between OS and CV events and mortality are very limited in PD patients.

## 5. PD Types and OS

Cueto-Manzano et al. reported on the effect of different PD types in serum and peritoneal dialysate concentrations of various inflammation markers [85]. In this crossover study, 11 high average or high transporters on CAPD were included. All patients were switched to nocturnal intermittent peritoneal dialysis (NIPD) and then to continuous cyclic peritoneal dialysis (CCPD). Plasma and dialysate levels of inflammation markers were measured at baseline, 10 days after start of NIPD, 10 days after commencement of CCPD and after a 12-month follow-up. NIPD was accompanied by a significant decrease in plasma levels of CRP, IL-6, and TNF-a, compared to CAPD and CCPD showed a tendency to increase all serum inflammation markers similarly to CAPD. The beneficial effect of NIPD in the inflammation status seemed to be independent of local inflammation of the peritoneum and might be attributed to peritoneal resting.

## 6. OS Effects on Peritoneal Membrane

Several investigators have suggested that the release of oxidation products may cause structural and functional alterations of the peritoneal membrane and subsequently loss of ultrafiltration. Gunal et al. studied the possible effect of OS abrogation on peritoneal membrane alterations induced by hypertonic PD solution in rats. Three groups of rats were followed for a 4-week period: the control group that received no treatment at all, the group that received hypertonic dextrose PD solution, and the group that received hypertonic solution plus trimetazidine intraperitoneally. The hypertonic PD solution group presented significantly altered morphology of the peritoneal membrane (increased thickness and neoangiogenesis) compared to the control group. Likewise, all the peritoneal function tests (ultrafiltration volume, one-hour peritoneal equilibration test, glucose reabsorption, and dialysate to plasma urea ratio) were significantly skewed in the hypertonic PD solution group. Furthermore, increased serum levels of MDA and VEGF and reduced activity of GPx were found in the rats treated with the hypertonic dextrose solution. The trimetazidine group showed significantly lesser degree of neovascularization, lower levels of MDA, VEGF, and increased GPx activity compared to the hypertonic dextrose PD solution treated rats. Trimetazidine seemed to play a protective role in the peritoneal functions by inhibiting the development of OS within the peritoneal membrane [86]. Honda et al. showed that enhanced OS in PD contributes to excessive accumulation of AGEs and glucose degradation products [87]. Another study suggested that accumulation of ROS induced by PD solutions is mainly responsible for fibrosis of the peritoneum, membrane hyperpermeability, and subsequent loss of ultrafiltration. The authors found that PD solutions with low pH caused an almost immediate (within the first minute) iron release from transferrin. This released iron led to elevated TBARS production and enhanced protein carbonylation in the erythrocytic membrane. Furthermore, accumulation of iron was found in the peritoneum of patients undergoing chronic PD, which might predispose to peritoneal fibrosis [88].

Taking the above-mentioned studies into account, it may be suggested that chronic accumulation of oxidative products, induced by the high-glucose environment within the peritoneal membrane, may lead to peritoneal fibrosis and subsequently, loss of ultrafiltration.

## 7. Peritonitis and OS

NO is one of the most important regulators of vascular tone and therefore affects peritoneal permeability and ultrafiltration capacity in PD patients. Excessive NO production mediates damage of the peritoneal membrane and chronic inflammation [89]. Yang et al. showed that NO is overproduced during a peritonitis episode in CAPD patients. After 7 days of antibiotic treatment, dialysate/plasma ratio of NO was significantly reduced. Fungal peritonitis led to a 2.5-fold increase and bacterial peritonitis to a 5.1-fold increase in NO levels compared to stable peritonitis-free CAPD controls. Patients with refractory peritonitis had significantly higher NO levels that did not decrease despite treatment [90]. These results were confirmed by other investigators: Choi et al. showed that the dialysate/plasma ratio of NO concentrations is a strong marker assessing the severity of peritonitis in CAPD patients as well as the effectiveness of treatment [91]. Duranay et al. found excessive NO peritoneal formation in CAPD patients with peritonitis [92], while Davenport et al. showed a significant local intraperitoneal NO generation by mesothelial cells and transmigrating macrophages during CAPD-related peritonitis [93].

## 8. Residual Renal Function (RRF) and OS in PD Patients

Numerous studies have highlighted the strong linkage between preservation of RRF and CV morbidity and mortality in PD patients. The CANUSA study found that a 250 ml larger daily urine volume decreased the relative risk of all-cause mortality by 36% [94]. In another study, RRF was strongly associated with reduced lipid peroxidation status. Urinary Kt/V and total weekly Kt/V urea were strongly associated with serum lipid hydroperoxides and free MDA levels, after adjustment for sex, inflammation, and nutritional factors [95]. Furuya et al. showed that PD patients with preserved RRF (>300 ml/day) had significantly higher plasma levels of AOPPs compared to PD subjects with daily urine volume less than 300 ml per day. After a 12-month follow-up, AOPP serum levels in patients whose urine daily volume decreased below 300 ml/day increased significantly while they remained unchanged in patients who maintained RRF > 300 ml/day. Moreover, AOPP plasma levels correlated strongly and negatively with changes in residual creatinine clearance. Thus, the authors concluded that RRF preservation reduced OS in PD patients [96]. In line with these results, Morinaga et al. showed that circulating free radicals were significantly and inversely associated with RRF in stable PD patients. Every 250 ml reduction in daily urine volume correlated strongly with a 0.1 au increase in plasma concentration of free radicals [97]. Furthermore, Feldman et al. reported that oral intake of 1200 mg of NAC twice daily for a period of 4 weeks resulted in significant improvement of RRF in PD patients. In these patients, daily urine volume and residual renal Kt/V were significantly increased after administration of NAC for a month [98].

## 9. Antioxidant Administration in PD

Chronic inflammation status and endothelial damage derived from enhanced OS are crucial precursors of cardiovascular CV disease in ESRD patients. Both *in vitro* and *in vivo* [99] studies have repeatedly highlighted the protective effects of antioxidant supplementation against inflammation, atherogenesis, and CV events. PD is accompanied by significant loss of antioxidants. Therefore, administration of antioxidants, such as NAC, vitamin E, and vitamin C, has been suggested for PD patients (Table 2).

### 9.1. Vitamin E Supplementation and OS

Ando et al. randomized 16 CAPD patients to receiving either 1.8 gr of eicosapentaenoic acid (EPA) for 3 months or placebo and found that EPA supplementation significantly decreased plasma levels of ox-LDL and atherogenic remnant lipoproteins [100]. Islam et al. administered alpha-tocopherol (AT, 800 I.U. per day) in HD patients, PD patients, and healthy matched controls for a 12-week period. AT supplementation reduced the susceptibility of LDL to oxidation in both HD and PD groups. After AT intake, there was an increase in serum lipid-standardized AT (controls = 150%, HD = 145%, and PD = 217%) and LDL AT levels (controls = 94%, HD = 94%, and PD = 135%) indicating that the positive protective effects of vitamin E seemed greater in PD patients than in the other two groups [101]. A prospective randomized, double-blind placebo-controlled trial of treatment with statin and vitamin E in HD and PD patients showed that use of statin improved significantly the lipid profile and ox-LDL levels and thus might be protective of CV complications in these patients. Additional administration of vitamin E did not influence any lipid parameters but reduced significantly *in vitro* LDL oxidizability and might act synergistically with statin treatment [102]. Domenici et al. reported that supplementation of vitamin E in both PD and HD patients resulted in decrease of protein oxidation and reduction of OS-induced DNA damage [103]. Uzum et al. showed that 300 mg/day oral intake of AT resulted in decreased levels of erythrocyte osmotic fragility (a marker of erythrocyte resistance to hemolysis when exposed to OS) in PD patients [23]. Similar results were published by Boudouris et al. who found that oral supplementation of vitamin C+E in PD patients, significantly reduced all OS markers in blood, urine, and peritoneal fluid [15]. However, another open-label study in 17 PD patients showed no beneficial effect of oral vitamin E intake on serum levels of autoantibodies against ox-LDL [104].

### 9.2. Ascorbic Acid/Vitamin C Supplementation and OS

Several investigators showed that PD is accompanied by significant loss of the antioxidant ascorbic acid and therefore, supplementation of this vitamin could be beneficial [105]. Shah et al. reported that oral intake of ascorbic acid resulted in a 45% increase of serum ascorbic acid levels in chronic PD patients [106]. Another cross-sectional study in 56 chronic PD patients highlighted the strong positive association between plasma vitamin C and hemoglobin levels and suggested vitamin C administration for better anemia management in these patients [107]. Sundl et al. reported that serum levels of ascorbate were significantly decreased in stable PD patients not receiving any vitamin C supplementation. No significant difference was found in AT and carotenoid levels between PD patients and healthy controls, but duration of PD treatment was negatively associated with *β*-carotene plasma concentrations. Therefore, low-dose vitamin C supplementation and diet rich in carotenoids was suggested for chronic PD patients [24].

### 9.3. NAC Supplementation and OS

NAC is a strong free radical scavenger that activates cysteine and glutathione and reduces ROS effectively [108]. Due to its anti-inflammatory actions, NAC has been widely used for the preservation of renal function in both acute and chronic renal damage mediated by OS, such as contrast-induced nephropathy and hepatorenal syndrome [109–112]. In HD patients, several authors reported that NAC administration results in a significant reduction of oxidative biomarkers (MDA and AOPP levels) possibly through elevation of glutathione serum levels [113, 114]. *In vivo* and *in vitro* studies have showed that NAC antioxidant functions could be beneficial for preventing peritoneal membrane sclerosis in rats and may reduce the peritoneal formation of AGEs [115, 116]. Furthermore, NAC was shown to be more effective than resting of the peritoneum in preventing peritoneal dysfunction in animal models [116]. In agreement with these results, a placebo-controlled study in patients undergoing PD found that daily oral intake of 1200 mg NAC for 2 months reduced plasma levels of IL-6 compared to placebo [117]. *In vivo*, the catastrophic effect of OS on the peritoneal membrane may be avoided with the use of antioxidants, since NAC seemed to prevent the progressive structural and functional changes in the peritoneal membrane induced by ROS in rats [118]. NAC inhibits the formation of prooxidant molecules that are precursors of peritoneal membrane modification and peritoneal fibrosis [119]. It has been reported that NAC can be safely added in the PD solution since it remains stable and preserves its antioxidant capacity if stored in a stable 25°C temperature for 15 days [120].

There is some evidence supporting that supplementation of antioxidants such as carotenoids and vitamins C and E may reduce the oxidation process in PD patients and NAC administration may play a protective role against OS and peritoneal fibrosis.

## 10. Conclusions

The accelerated OS status that accompanies PD patients even in the first year after initiation of the method is mainly due to the composition of the PD solutions. Therefore, chronic accumulation of AGEs mainly due to the high glucose of the peritoneal fluids may lead to OS, inflammation, and chronic structural and functional deterioration of the peritoneal membrane which results in ultrafiltration loss, endothelial damage, and tissue injury. Although PD is a state of increased OS, it may be considered as a more biocompatible form of dialysis, regarding the production of prooxidative compounds, when compared to HD. The type of PD therapy seems to exert a direct effect on inflammation and OS of the peritoneum, with NIPD being probably the less harmful. Further, larger studies in this group of patients are needed to elucidate the possible role of OS in the pathogenesis of atherosclerosis and CV disease. Exogenous antioxidant supplementation may be beneficial for chronic PD patients, but in the light of the limited current evidence cannot be recommended in routine clinical practice. Considering the numerous, complex, interrelated pathophysiological mechanisms underlying the fibrosis of peritoneal membrane and the subsequent loss of ultrafiltration, accelerated OS may emerge as a novel molecular pathway underlying the harmful effects of long-term PD on the peritoneal membrane.

## Figures and Tables

**Table 1:** Comparison of OS status in HD versus PD patients.

Study (ref.)	Year	OS biomarker	Patients	Results
Similar OS status in HD and PD (6 studies)				
Filiopoulos et al. [42]	2009	Serum TAC Serum SOD	20 HD 11 PD	Similar in HD and PD
Ahmadpoor et al. [43]	2009	Serum GPx activity Serum TAC Serum GSH RBC GSH	30 HD 12 CAPD	Similar in HD and CAPD
Mekki et al. [44]	2010	Serum TBARS Serum SOD Serum GPx Serum CAT	20 HD 20 PD	Similar in HD and PD
Castoldi et al. [41]	2010	Serum 8-OHdG Serum ox-LDL	51 HD 17 PD	Similar in HD and PD
Samouilidou et al. [40]	2012	Serum ox-LDL	31 HD 24 PD	Similar in HD and PD
Mehmetoglu et al. [45]	2012	Serum TAC Serum coenzyme Q10	38 HD 41 CAPD	Similar in HD and CAPD

Lower OS status in HD than in PD (3 studies)				
Taylor et al. [47]	1992	Serum MDA	20 HD 18 CAPD	Higher in CAPD versus HD
		Serum SOD		Similar in CAPD and HD
McGrath et al. [48]	1995	Serum MDA	20 HD 20 CAPD	Higher in CAPD versus HD
Al-Hweish et al. [46]	2010	Serum MPO	56 HD 28 CAPD	Higher in CAPD versus HD

Higher OS status in HD than PD (24 studies)				
Pastor et al. [72]	1993	Serum AT levels (antioxidant)	18 HD 14 CAPD	Lower in HD versus CAPD
Ross et al. [68]	1997	Serum cysteine	20 HD 20 PD	Higher in HD versus PD
		Serum glutathione		Similar in HD and PD
Zima et al. [71]	1998	Serum. RBC and whole blood Zn, Cu, and Se (antioxidants)	36 HD 18 CAPD	Lower in HD versus CAPD
Kielstein et al. [50]	1999	Serum ADMA	43 HD 37 PD	Higher in HD versus PD
Takayama et al. [69]	2001	Serum glutathionyl hemoglobin	30 HD 10 CAPD	Higher in HD versus CAPD
Donate et al. [66]	2002	Serum carbonyl proteins	42 HD 21 PD	Higher in HD versus PD
Lim et al. [51]	2002	Serum F2-isoprostanes	35 HD 30 CAPD	Higher in HD versus CAPD
Usberti et al. [54]	2002	Serum ROS, MDA, and TAC	55 HD 16 PD	Higher in HD versus PD
		Serum AT (antioxidant)		Lower in HD versus PD
Yonova et al. [59]	2004	Serum MDA Serum ox-LDL	22 HD 22 CAPD	Higher in HD versus PD
		Serum vitamin E Serum vitamin C Serum GPx		Lower in HD versus PD
Lucchi et al. [53]	2005	RBC GSH	23 HD 15 CAPD	Higher in HD versus PD
Sela et al. [55]	2005	Rate of superoxide release from activated neutrophils	30 HD 30 CAPD	Higher in HD versus CAPD
Morimoto et al. [56]	2005	Serum ADMA	31 HD 43 PD	Higher in HD versus PD
Pawlak et al. [57]	2007	Serum Cu/Zn SOD Serum VEGF	25 HD 45 PD	Higher in HD versus PD
Mitrogianni et al. [65]	2009	Serum carbonyl proteins	25 HD 21 PD	Higher in HD versus PD
De Rojas and Mateo [73]	2009	RBC SOD activity RBC CAT activity (antioxidants)	16 HD 11 CAPD	Lower in HD versus CAPD
Kayabasi et al. [58]	2010	Serum MDA and GPx	32 HD 39 CAPD	Similar in HD versus CAPD
		Serum SOD (antioxidant)		Lower in HD versus CAPD
Guoa et al. [60]	2011	Serum TBARS, carbonyl compounds	20 HD 20 PD	Higher in HD versus PD
		GPx and CAT activity (antioxidant markers)		Lower in HD versus PD
		Serum Zn, Se (antioxidants)		Lower in HD versus PD
Zhou et al. [61]	2012	Serum AOPPs	1539 HD 556 CAPD	Higher in HD versus PD
Marques de Mattos et al. [62]	2012	Serum AOPPs	48 HD 20 CAPD	Higher in HD versus PD
Capusa et al. [64]	2012	Serum TBARS	16 HD 17 CAPD	Higher in HD versus PD
		Serum TAC (antioxidant marker)		Similar in HD and PD
Puchades et al. [52]	2013	Serum carbonyl proteins Serum F2-isoprostanes Serum 8-OHdG	30 HD 31 PD	Higher in HD versus PD
		Serum GPx Serum GSH Serum CAT, SOD (antioxidants)		Lower in HD versus PD
Marques de Mattos et al. [63]	2014	Serum AOPPs Serum AGEs	21 HD 19 PD	Higher in HD versus PD
		Serum vitamin C+A+E (antioxidants)		Similar in HD and PD
Xu et al. [67]	2015	Serum8-OHdG	220 HD 83 PD	Higher in HD versus PD
Stępniewska et al. [70]	2016	Platelet CAT activity (antioxidant marker)	37 HD 23 PD	Lower in HD versus PD (0.82 in PD versus 0.52 before HD and 0.35 after HD)

OS: oxidative stress; HD: hemodialysis; PD: peritoneal dialysis; ox-LDL: oxidized low-density lipoprotein; 8-OHdG: 8-hydroxy-2′-deoxyguanosine; TAC: total antioxidant capacity; SOD: superoxide dismutase; MPO: myeloperoxidase; CAPD: continuous ambulatory peritoneal dialysis; MDA: malondialdehyde; ADMA: asymmetric dimethylarginine; RBC: red blood cell; GSH: reduced glutathione; ROS: reactive oxygen species; AT: alpha-tocopherol; SOD: superoxide dismutase; VEGF: vascular endothelial growth factor; GPx: glutathione peroxidase; TBAR: thiobarbituric acid-reactive substances; AOPPs: advanced oxidation protein products; CAT: catalase; Zn; zinc; Cu; copper; Se: selenium.

**Table 2:** Effect of antioxidant supplementation on PD patients.

Study	Year	Patient modality	Antioxidant	Study period	Results
Shah et al. [106]	1991	7 PD	100 mg ascorbic acid/day	4 weeks	↑ 45% in serum ascorbic acid levels
Ando et al. [100]	1999	16 CAPD	1.8 gr EPA/day	12 weeks	↓ serum ox-LDL levels
Islam et al. [101]	2000	17 PD	800 IU AT/day	12 weeks	↓ susceptibility of LDL oxidation ↑ 217% serum lipid-standardized AT ↑ 135% serum LDL AT levels
O' Byrne et al. [104]	2001	17 PD	800 IU AT/day	12 weeks	↑ serum LDL AT levels No effect on autoantibodies to ox-LDL
Diepeveen et al. [102]	2005	21 PD	40 mg atorvastatin/day	12 weeks	↓ serum ox-LDL levels Improved lipid profile No effect on in vitro LDL oxidizability
			800 IU AT/day	12 weeks	No effect on serum ox-LDL levels No effect on lipid profile ↓ in vitro LDL oxidizability
Domenici et al. [103]	2005	22 PD	300 mg AT x3/week	4 weeks	↑ 50% serum vitamin E levels ↓ protein oxidation ↓ OS-induced DNA damage (serum levels 8-OHdG)
Uzum et al. [23]	2006	13 PD	300 mg AT/day	20 weeks	↑ serum vitamin E levels ↓ erythrocyte osmotic fragility
Nascimento et al. [117]	2010	30 PD	1200 mg NAC/day	8 weeks	↑ serum NAC levels (2.6 to 24.8 mmol/l) ↓ serum IL-6 levels(9.4 to 7.6 pg/ml)
Boudouris et al. [15]	2013	20 CAPD	250 mg vitamin C/day 400 IU AT/day	8 weeks	↑ serum, urine, and peritoneal fluid TAC levels ↓ serum, urine, and peritoneal fluid MDA levels

EPA: eicosapentaenoic acid; NAC: n-acetylcysteine; IL-6: interleukin-6.

## Abbreviations

8-OHdG: 8-hydroxy-2′-deoxyguanosine
ADMA: Asymmetric dimethylarginine
AGEs: Advanced glycation end products
AOPPs: Advanced oxidation protein products
AT: Alpha-tocopherol
CAPD: Continuous ambulatory peritoneal dialysis
CCPD: Continuous cyclic peritoneal dialysis
CIMT: Carotid intima-media thickness
CKD: Chronic kidney disease
CRP: C-reactive protein
CV: Cardiovascular
DNA: Deoxyribonucleic acid
DM: Diabetes mellitus
EPA: Eicosapentaenoic acid
ESRD: End-stage renal disease
GPx: Glutathione peroxidase
GSH: Glutathione
GSSG: Oxidized glutathione
HD: Hemodialysis
HDL: High-density lipoprotein
IL-6: Interleukin -6
LDL: Low-density lipoprotein
MDA: Malondialdehyde
MPO: Myeloperoxidase
NAC: N-acetylcysteine
NIPD: Nocturnal intermittent peritoneal dialysis
NO: Nitric oxide
NOS: Nitric oxide synthase
OS: Oxidative stress
Ox-LDL: Oxidized LDL
PD: Peritoneal dialysis
RBC: Red blood cell
ROOH: Lipid hydroperoxides
ROS: Reactive oxygen species
RRF: Residual renal function
SOD: Superoxide dismutase
TAC: Total antioxidant capacity
TBARS: Thiobarbituric acid-reactive substances
TGF: Transforming growth factor
TNF: Tumor necrosis factor
VEGF: Vascular endothelial growth factor.
